# Comparing the Oncological Outcomes of Cryoablation vs. Radical Prostatectomy in Low-Intermediate Risk Localized Prostate Cancer

**DOI:** 10.3389/fonc.2020.01489

**Published:** 2020-08-26

**Authors:** Xiao-xiao Guo, Sheng-jie Liu, Miao Wang, Hui-min Hou, Xuan Wang, Zhi-peng Zhang, Ming Liu, Jian-ye Wang

**Affiliations:** ^1^Department of Urology, National Center of Gerontology, Institute of Geriatric Medicine, Beijing Hospital, Chinese Academy of Medical Science, Beijing, China; ^2^Graduate School of Peking Union Medical College, Beijing, China

**Keywords:** cryoablation, radical prostatectomy, propensity score matching, survival outcomes, SEER database

## Abstract

**Purpose:** To compare the oncologic outcomes of cryoablation (CA) and radical prostatectomy (RP) in patients with low- and intermediate-risk localized prostate cancer (PCa).

**Materials and Methods:** PCa patients who received CA or RP between 2004 and 2015 were identified from the Surveillance, Epidemiology, and End Results database. Multivariable Cox proportional hazard analysis was used to compare the prostate cancer-specific survival (CSS) and overall survival (OS). We conducted 1:3 propensity score matching and adjusted standardized mortality ratio weighting (SMRW) to balance the clinicopathological characteristics.

**Results:** Ninety-seven thousand seven hundred eighty-three patients were identified after preliminary screening. After matching, the CA and RP groups included 1,942 and 5,826 patients and had median follow-up periods of 85 and 72 months, respectively. CA had lower CSS and OS rates (hazard ratio [HR], 2.07; *P* = 0.007; HR, 2.09; *P* < 0.001, respectively) than did RP, which was consistent in the SMRW model (CSM: HR, 2.66; *P* < 0.001; OS: HR, 2.29; *P* < 0.001). The 10-years CSS and OS for CA vs. RP were 98.1 vs. 99.2% and 61.3 vs. 79.9%, respectively.

**Conclusions:** In patients with low- to intermediate-risk localized PCa, CA had lower CSS rates than did RP. However, the high 10-years CSS rates indicated that CA could be an option for those who are not RP candidates. Further high-quality trials are needed to confirm and expand our findings.

## Introduction

The screening strategies of prostate cancer (PCa) have been used to identify men at an earlier stage with small tumor volumes (1, 2), resulting in controversy about active surveillance and radical treatments. Ablation, a minimally invasive procedure, can serve as a compromise between radical and conservative treatment (3). Previous studies have proven the safety of ablation therapy (4, 5) and reported a good oncological efficacy of it in patients with localized PCa (6, 7).

Cryoablation (CA), which uses extremely cold temperature to induce tumor necrosis, has been one of the most commonly used ablation modalities. Some single-arm case series showed that CA has a favorable short-intermediate term oncological efficacy in patients with clinically low-intermediate risk PCa (8, 9). Furthermore, other studies comparing radical prostatectomy (RP) and CA reported comparable oncological outcomes between the two interventions (10, 11). However, given the lack of comparative study of CA vs. RP with mid-long follow-up period, significant uncertainties remain in considering CA as an alternative strategy to radical prostatectomy (RP) for localized PCa. Therefore, the present study aimed to validate the efficacy of CA for low-intermediate risk localized PCa compared with RP.

## Materials and Methods

### Study Population

**Figure 1:**
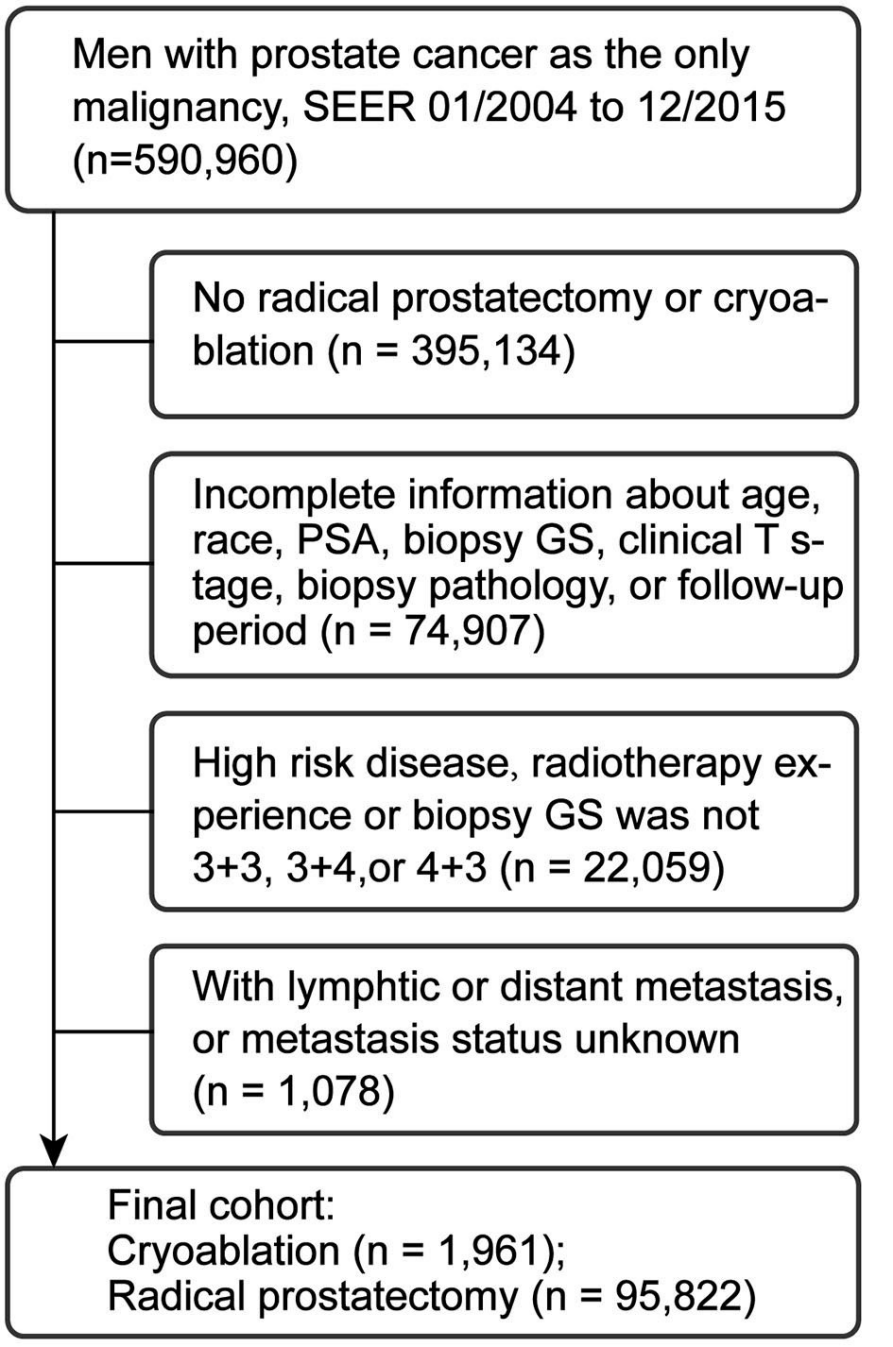
Study cohort selection. PSA, prostate specific antigen; PCa, prostate cancer; SEER, surveillance, epidemiology, and end results database; GS, Gleason score.

We used the Surveillance, Epidemiology, and End Results (SEER) database to identify patients with PCa from 2004 to 2015. The SEER programme of the US National Cancer Institute was used as the data source for the present study. The 17th SEER tumor registries encompass ~26% of the US population. The SEER programme collects information on cancer incidence, prevalence, survival, and mortality of cancer patients. This programme has 98% completeness in ascertaining cases. In total, 590,960 patients with PCa as the only malignancy were initially screened. Patients who had not received RP or CA (*n* = 395,134) or with incomplete clinicopathological data (*n* = 74,907) were primarily excluded. Then, patients with high-risk disease, radiotherapy experience, biopsy Gleason Score (GS) other than 3 + 3, 3 + 4, or 4 + 3, or metastasis were also excluded (Figure 1). Finally, 97,783 patients with clinical T1c–T2b tumor, prostate specific antigen (PSA) ≤20 ng/ml before treatment, and biopsy GS ≤7 (3 + 3, 3 + 4, and 4 + 3) were included.

### Propensity Score Matching

We conducted 1:3 propensity score matching (PSM) with specified caliper distances of 0.5 to diminish residual and selection bias. Age, PSA value, race, clinical tumor stage, biopsy GS, and year of diagnosis were adjusted by the logistic regression model to calculate the propensity score. The standardized mean difference (SMD) and propensity score (PS) density plot were used to evaluate the matching efficiency.

### Statistical Analysis

The mean [standard deviation (SD)] was used to report continuous variables, and frequency and proportion were used for categorical variables. Clinicopathological features of both groups were compared by the Kruskal-Wallis, Wilcoxon rank-sum, or Fisher's exact tests. The hazard ratio (HR) with 95% confidence interval (CI) for CSS and OS of both groups were calculated by using multivariable Cox proportional hazards model. The standardized mortality ratio weighting (SMRW) model that unified the distribution of the risk factors of both groups was used to confirm the robustness of the results. In addition, subgroup analyses were conducted in terms of D'Amico risk group (low- and intermediate-risk), clinical tumor stage (T1c and T2a−2b), preoperative PSA level (≤10, and 10.1–20 ng/ml), and biopsy GS (3 + 3, 3 + 4, and 4 + 3). All analyses were performed by R version 3.6.1 software (The R Foundation for Statistical Computing, Vienna, Austria; www.r-project.org) with the R packages “survminer” and “Matching.” All statistical testing was two-sided with significance considered at a *P*-value of 0.05.

## Results

As Table 1 shows, the present study included 97,783 patients. Among these, 95,822 patients received RP, while 1,961 received CA. Before PSM, all key variables differed significantly between the RP and CA groups (all *P* < 0.001). After PSM, the CA and RP groups included 1,942 and 5,826 cases, respectively. The two groups were well-balanced except for the race distribution, as there were more African Americans in the CA group (10.7 vs. 16.5%; *P* < 0.001). The mean age and PSA were 68.6 vs. 68.2 years and 6.7 vs. 6.8 ng/ml for CA vs. RP (*p* = 0.072 and 0.696, respectively). The median follow-up periods of CA and RP were 84 and 83 months, respectively. All the SMD values in the PSM and SMRW cohorts were <10% and were far less than in the unmatched cohort (Supplemental Figure 1). The PS distribution of the CA and RP group in PSM and SMRW cohorts were highly consistent (Supplemental Figure 2).

**Table 1:** Baseline demographic and clinicopathologic characteristics sort by interventions before and after propensity score matching.

	**Before matching**			**After matching**		
	**RP (*N* = 95,822)**	**CA (*n* = 1,961)**	***P*-value**	**RP (*n* = 5,826)**	**CA (*n* = 1,942)**	***P*-value**
**Age, year Mean (SD)**	60.2 (7.1)	68.7 (7.6)	<0.001	68.2 (6.97)	68.6 (7.4)	0.072
**Race**, ***n*** **(%)**			<0.001			<0.001
Caucasian	77,288 (80.7)	1,518 (77.4)		4,626 (79.4)	1,501 (77.3)	
African	12,685 (13.2)	321 (16.4)		623 (10.7)	320 (16.5)	
Other	4,894 (5.1)	96 (4.9)		513 (8.8)	95 (4.9)	
Unknown	955 (1.0)	26 (1.3)		64 (1.7)	26 (1.3)	
**PSA, ng/mL Mean (SD)**	6.3 (3.1)	6.7 (3.4)	<0.001	6.8 (3.4)	6.7 (3.3)	0.696
<4	14,325 (14.9)	263 (13.4)		742 (12.7)	262 (13.5)	
4–10	71,011 (74.1)	1,425 (72.7)		4,288 (73.6)	1,410 (72.6)	
10.1–20	10,486 (10.9)	273 (13.9)		796 (13.7)	270 (13.9)	
**Clinical T stage**, ***n*** **(%)**			<0.001			0.616
T1c	85,531 (89.3)	1,658 (84.5)		4,880 (83.8)	1,645 (84.7)	
T2a	7,880 (8.2)	203 (10.4)		639 (11.0)	201 (10.4)	
T2b	2,411 (2.5)	100 (5.1)		307 (5.3)	96 (4.9)	
**Biopsy GS**, ***n*** **(%)**			<0.001			0.937
3 + 3	43,665 (45.6)	952 (48.5)		2,842 (48.8)	939 (48.4)	
3 + 4	39,503 (41.2)	695 (35.4)		2,051 (35.2)	692 (35.6)	
4 + 3	12,654 (13.2)	314 (16.0)		933 (16.0)	311 (16.0)	
**Year of diagnosis**, ***n*** **(%)**			<0.001			0.401
2004–2007	29,133 (30.4)	767 (39.1)		2,202 (37.8)	765 (39.4)	
2008–2011	37,502 (39.1)	749 (38.2)		2,220 (38.1)	728 (37.5)	
2012–2015	29,187 (30.5)	445 (22.7)		1,404 (24.1)	449 (23.1)	
**D'Amico risk group** ***n*** **(%)**			0.422			0.831
Low	36,081 (37.7)	721 (36.8)		2,399 (42.1)	805 (41.5%)	
Intermediate	59,741 (62.3)	1,240 (63.2)		3,427 (58.8)	1,137 (58.5%)	
**Follow-up, month (median [IQR])**	78 [48, 111]	84 [53, 113]		83 [52, 115]	84 [53, 113]	

*RP, radical prostatectomy; CA, cryoablation; PSA, prostate specific antigen; GS, Gleason score; SD, standard deviation; IQR, interquartile range*.

**Figure 2:**
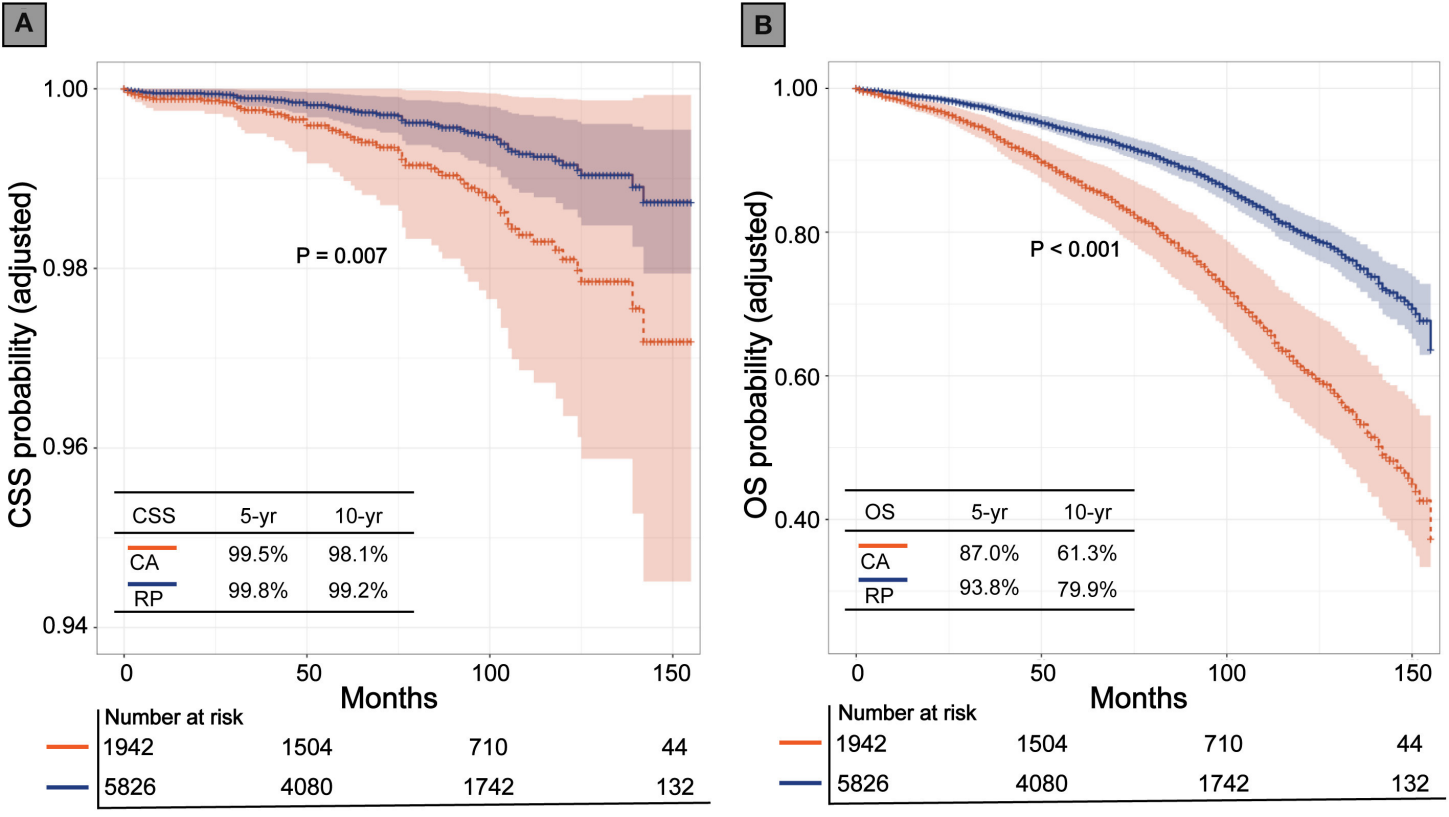
Kaplan-Meier survival curves of cryoablation (CA) vs. radical prostatectomy (RP) after propensity score matching. **(A)** Cancer specific survival (CSS); **(B)** Overall survival (OS).

Before matching, the multivariable Cox regression model showed that RP was associated with higher CSS and OS rates (HR, 2.32; 95% CI, 1.54–3.50; *P* < 0.001), (HR, 2.34; 95% CI, 2.07–2.64; *P* < 0.001), respectively. After PSM, CA still showed inferior CSS and OS to that of RP (HR, 2.07; 95% CI, 1.22–3.51; *P* = 0.007; *P* < 0.001), (HR, 2.09; 95% CI, 1.80–2.44; *P* < 0.001; respectively). The Kaplan-Meier (K-M) survival curves of CSS and OS are presented in Figure 2. In patients who received CA and RP, the 5- and 10-years CSS rates were 99.5 vs. 99.8% and 98.1 vs. 99.2%, respectively; the 5- and 10-years OS rates were 87.0 vs. 93.8% and 61.3 vs. 79.9%, respectively. Consistently, the adjusted SMRW model supported the inferior survival outcomes of CA (CSS: HR, 2.66; 95%CI, 1.27–5.59; *P* = 0.010; OS: HR, 2.29; 95%CI, 1.85–2.83; *P* < 0.001). All the results of the Cox regression analyses are summarized in Table 2.

**Table 2:** Effect of cryoablation vs. radical prostatectomy on oncologic outcomes.

**Cancer-specific survival**	**HR**	**95%CI**	***P*-value**
In adjusted non-matched cohort	2.32	1.54–3.50	<0.001
In adjusted matched cohort	2.07	1.22–3.51	0.007
In adjusted SMRW model	2.66	1.27–5.59	0.010
**Overall survival**	**HR**	**95%CI**	***P*****-value**
In adjusted non-matched cohort	2.34	2.07–2.64	<0.001
In adjusted matched cohort	2.09	1.80–2.44	<0.001
In adjusted SMRW model	2.29	1.85–2.83	<0.001

*HR, hazard ratio; CI, confidence interval; SMRW, standardized mortality ratio weighting*.

**Figure 3:**
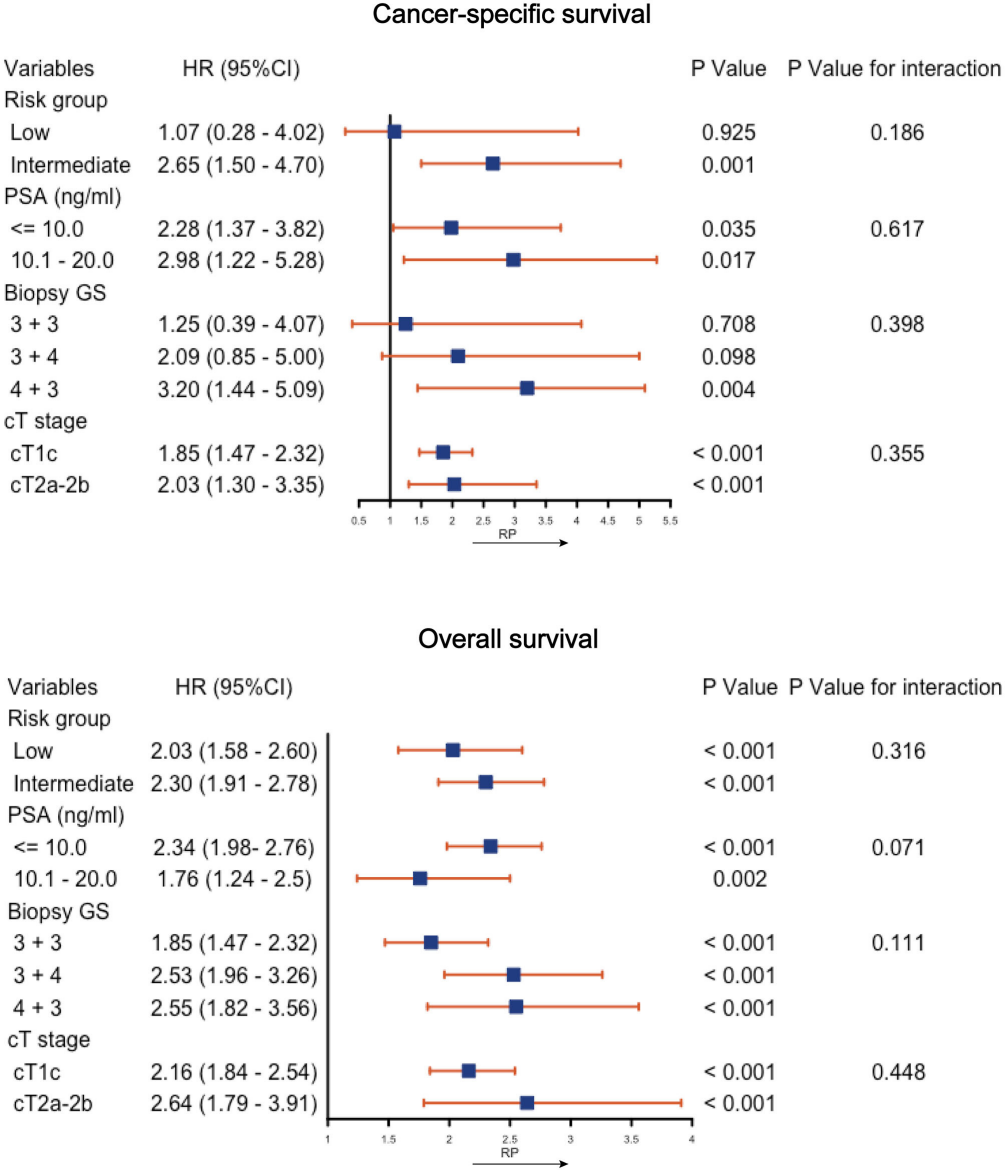
Subgroup analysis of cancer-specific survival and overall survival by cryoablation vs. radical prostatectomy (RP). PSA, prostate specific antigen; GS, Gleason score; CI, confidence interval; HR, hazard ratio.

Figure 3 shows the Forest plots of the subgroup analysis. The OS of the RP was generally better than that of CA in all the subgroups. The CSS was similar between the two interventions in the subgroups of low risk (HR, 1.25; 95% CI, 0.39–4.07; *P* = 0.708), GS 3 + 3 (HR, 1.25; 95% CI, 0.39–4.07; *P* = 0.708), or GS 3 + 4 (HR, 2.09; 95% CI, 0.85–5.00; *P* = 0.098), while CA was associated with a lower CSS in the remaining subgroups. Tests for interaction revealed no significant interaction between the variables and the effect of interventions on the CSS (all *P*-values for interactions >0.05). Supplemental Figure 3 shows the K-M CSS curves and estimated 5- and 10-years CSS rates of each subgroup.

## Discussion

The present study, involving patients with low- and intermediate-risk localized PCa, compared mid- to long-term oncological outcomes between CA and RP. The results showed that CA had a lower CSS and OS than RP. The 10-years CSS of CA in all low-intermediate risk, low-risk, and intermediate-risk patients were 98.1, 99.3, and 98.0%, respectively.

Some studies have reported the oncological outcomes after CA for localized PCa. Lian et al. (8) in their series of 41 patients with low- and intermediate-risk PCa reported 10% (4/40) treatment failure (at least one positive biopsy in the treated lobe, or biochemical recurrence) rates after focal CA with a median follow-up of 63 months. Oishi et al. (9) collected data from 160 consecutive men who underwent hemi-gland CA for localized low-, intermediate-, or high-risk PCa and reported 62% biochemical-free survival rates and 89% clinically significant PCa-free rates. Nevertheless, these single-arm studies did not account for the differences in oncological outcomes between the CA and other interventions. Bahn et al. (11) who compared the oncological outcomes between 36 focal CA patients and 36 matched RP patients, reported a similar risk for the need for salvage treatment between the two interventions within a 3.7-years follow-up period. However, the CSS and OS were not reported in their study. The small sample size and short follow-up period of the study may be too low to show significance. Our study revealed that patients who received CA had approximately a 2-fold higher risk of CSM than did those who received RP. The results of the SMRW cohort supported the validity of the results. Garcia-Barreras et al. (10) also reported an inferior efficacy of CA. They compared the oncological outcomes between 236 partial gland CA patients and 472 matched robot-assisted RP patients. They found that CA was associated with a higher PSA nadir and time to PSA nadir. Patients with CA had a 6-fold higher risk for salvage treatment. In another study, Elkjær et al. (12) retrospectively compared the oncological outcomes of 39 PCa patients received whole-gland CA while 350 patients received RP. The results showed a higher recurrence risk in CA group. The primary reason for the inferior oncological outcomes of CA could be the multifocality of PCa (13), especially in patients with focal CA. Lian et al. (8) reported a 12.5% (5/40) rate of positive biopsy in the contralateral lobe during follow-up biopsy. In addition, Bahn et al. (11) reported a 16% rate of positive biopsy in the contralateral lobe in 48 patients who received CA and at least one post-CA biopsy. Moreover, there are two other possible explanations. The cryotherapy does not create a uniform frozen zone but lowest temperature in the center and higher toward the periphery (14, 15). Lightly damaged cancer cells can repair themselves and survive. The close anatomical relations between the prostate and the rectum and nerve vascular bundle makes it hard to obtain a safe margin in some cases, which results in remaining tumor tissue. The progress of untreated or remained lesions ultimately leads to poorer oncological outcomes of the CA group.

The criteria for CA was low-risk (GS 3 + 3) disease with a life expectancy of at least 10 years. In the recent consensus, patients with a Gleason 7 (3 + 4) lesion were also considered as candidates (16–18). Our results indicated that in the low-risk and GS 3 + 4 subgroups, no statistical difference in CSS rates existed between CA and RP. Nevertheless, the test for interaction suggested no significant interaction between the risk stratification or GS score and the effect of treatment on the CSS (*P* = 0.186, 0.398, respectively). The comparable efficiency between the two groups in these subgroups may be due to the few deaths during the short follow-up period. Therefore, we considered these subgroup analysis results consistent with the overall results. In a report from an international Delphi consensus project (19), 80% of the panel of experts agreed to treat GS 4 + 3 disease up to 0.5 ml with focal therapy. Our results showed a lower CSS rates of CA compared to that of RP in the GS 4 + 3 subgroup. Moreover, the CSS differences between the two interventions in GS 4 + 3 subgroup (83.4 vs. 94.3%) was higher than that in other subgroups. This is understandable. Higher Gleason pattern is a crucial predictor for capsular perforation and seminal vesicle invasion (20, 21), which further increases the risk of incomplete ablation, ultimately causing poorer oncological outcomes.

Recently, a randomized controlled trial (22) compared focal ablation and AS in patients with low-risk PCa. The short-term results showed significantly reduced treatment failure in focal ablation group. The latest report of the prostate cancer intervention vs. observation trial (PIVOT) (23) showed that surgery was associated with decreased all-cause mortality and increased years of life gained compared with AS. These reports indicate that the active interventions may benefit patients. Although CA (an active intervention) had lower CSS than did RP in low-intermediate risk patients, its 10-years CSS in low- and intermediate-risk patients were as high as 99.3 and 98.0%, respectively, making it an excellent option for those who cannot tolerate RP.

The present study has some clinical implications. The results showed that compared with RP, CA had inferior CSS and OS in patients with low-intermediate risk PCa. This suggested that CA should not be the first choice in cases wherein a cure is the top priority. However, in cases wherein the top priority is maintaining the quality of life or for patients ineligible for RP, CA is worth considering as it provides effective mid-long CSS. Our study has several limitations as well. First, the precise extension of ablation was not defined owing to the nature of the SEER. Hence, we could not perform further subgroup analyses regarding different ablation extensions vs. RP. Although previous studies with short–mid follow-up reported similar oncological outcomes between different ablation extensions (24, 25), it is worthwhile to note that the conclusions may change with the extension of follow-up. Second, the much greater difference in OS compared to that in CSS suggested that non-cancer factors are the leading cause of patient death. However, the impact of CA on OS cannot be determined with the present study because we cannot assess the frailty and comorbidity of patients. Third, although we excluded patients with radiotherapy experience, there were still some patients who received salvage CA, which could potentially affect prognosis.

## Conclusion

In patients with low-intermediate localized PCa, CA had lower CSS than did RP, suggesting that RP provided better tumor control than did CA. However, for patients who are not RP candidates, CA is worth considering due to positive 10-years CSS outcomes. There is need for high-quality trials to compare the oncological outcomes of different extensions of ablation with those of radical treatments and clarify the effect of CA on OS in comparison with radical treatments.

## Data Availability Statement

Publicly available datasets were analyzed in this study. This data can be found here: Surveillance, Epidemiology, and End Results (SEER) database (https://seer.cancer.gov/).

## Author Contributions

JW and ML: conception of the work. XG: acquisition and interpretation of the data. XG, SL, and MW: drafting the manuscript. HH, XW, and ZZ: critical revision for important intellectual content. XG and SL contributed equally to the work. All authors contributed to the article and approved the submitted version.

## Conflict of Interest

The authors declare that the research was conducted in the absence of any commercial or financial relationships that could be construed as a potential conflict of interest.

## References

[1] Mouraviev V Mayes JM Polascik TJ. Pathologic basis of focal therapy for early-stage prostate cancer. Nat Rev Urol. (2009) 6:205–15. 10.1038/nrurol.2009.29 19352395

[2] Polascik TJ Mayes JM Sun L Madden JF Moul JW Mouraviev V. Pathologic stage T2a and T2b prostate cancer in the recent prostate-specific antigen era: implications for unilateral ablative therapy. Prostate. (2008) 68:1380–6. 10.1002/pros.20804 18543281

[3] Eggener SE Scardino PT Carroll PR Zelefsky MJ Sartor O Hricak H. Focal therapy for localized prostate cancer: a critical appraisal of rationale and modalities. J Urol. (2007) 178:2260–7. 10.1016/j.juro.2007.08.072 17936815

[4] Valerio M Cerantola Y Eggener SE Lepor H Polascik TJ Villers A. New and established technology in focal ablation of the prostate: a systematic review. Eur Urol. (2017) 71:17–34. 10.1016/j.eururo.2016.08.044 27595377

[5] Jung JH Risk MC Goldfarb R Reddy B Coles B Dahm P. Primary cryotherapy for localised or locally advanced prostate cancer. Cochrane Database Syst Rev. (2018) 5:CD005010. 10.1002/14651858.CD005010.pub3 29845595 PMC6494517

[6] Albisinni S Aoun F Bellucci S Biaou I Limani K Hawaux E. Comparing high-intensity focal ultrasound hemiablation to robotic radical prostatectomy in the management of unilateral prostate cancer: a matched-pair analysis. J Endourol. (2017) 31:14–9. 10.1089/end.2016.0702 27799004

[7] Guillaumier S Peters M Arya M Afzal N Charman S Dudderidge T. A multicentre study of 5-year outcomes following focal therapy in treating clinically significant nonmetastatic prostate cancer. Eur Urol. (2018) 74:422–9. 10.1016/j.eururo.2018.06.006 29960750 PMC6156573

[8] Lian H Zhuang J Yang R Qu F Wang W Lin T. Focal cryoablation for unilateral low-intermediate-risk prostate cancer: 63-month mean follow-up results of 41 patients. Int Urol Nephrol. (2016) 48:85–90. 10.1007/s11255-015-1140-8 26531063

[9] Oishi M Gill IS Tafuri A Shakir A Cacciamani GE Iwata T. Hemigland cryoablation of localized low, intermediate and high risk prostate cancer: oncologic and functional outcomes at 5 years. J Urol. (2019) 202:1188–98. 10.1097/JU.0000000000000456 31347953 PMC9235523

[10] Garcia-Barreras S Sanchez-Salas R Sivaraman A Barret E Secin F Nunes-Silva I. Comparative analysis of partial gland ablation and radical prostatectomy to treat low and intermediate risk prostate cancer: oncologic and functional outcomes. J Urol. (2018) 199:140–6. 10.1016/j.juro.2017.08.076 28823768

[11] Bahn D de Castro Abreu AL Gill IS Hung AJ Silverman P Gross ME. Focal cryotherapy for clinically unilateral, low-intermediate risk prostate cancer in 73 men with a median follow-up of 3.7 years. Eur Urol. (2012) 62:55–63. 10.1016/j.eururo.2012.03.006 22445223

[12] Elkjaer MC Borre M. Oncological outcome after primary prostate cryoablation compared with radical prostatectomy: a single-centre experience. Scand J Urol. (2014) 48:27–33. 10.3109/21681805.2013.792102 23597178

[13] Masterson TA Cheng L Koch MO. Pathological characterization of unifocal prostate cancers in whole-mount radical prostatectomy specimens. BJU Int. (2011) 107:1587–91. 10.1111/j.1464-410X.2010.09849.x 21062398

[14] Gage AA Baust JG. Cryosurgery for tumors. J Am Coll Surg. (2007) 205:342–56. 10.1016/j.jamcollsurg.2007.03.007 17660083

[15] Gage AA Baust JG. Cryosurgery for tumors–a clinical overview. Technol Cancer Res Treat. (2004) 3:187–99. 10.1177/153303460400300212 15059025

[16] van den Bos W Muller BG Ahmed H Bangma CH Barret E Crouzet S. Focal therapy in prostate cancer: international multidisciplinary consensus on trial design. Eur Urol. (2014) 65:1078–83. 10.1016/j.eururo.2014.01.001 24444476

[17] Langley S Ahmed HU Al-Qaisieh B Bostwick D Dickinson L Veiga FG. Report of a consensus meeting on focal low dose rate brachytherapy for prostate cancer. BJU Int. (2012) 109:7–16. 10.1111/j.1464-410X.2011.10825.x 22239224

[18] van der Poel HG van den Bergh RCN Briers E Cornford P Govorov A Henry AM. Focal therapy in primary localised prostate cancer: the European Association of Urology Position in 2018. Eur Urol. (2018) 74:84–91. 10.1016/j.eururo.2018.01.001 29373215

[19] Tay KJ Scheltema MJ Ahmed HU Barret E Coleman JA Dominguez-Escrig J. Patient selection for prostate focal therapy in the era of active surveillance: an International Delphi Consensus Project. Prostate Cancer Prostatic Dis. (2017) 20:294–9. 10.1038/pcan.2017.8 28349978

[20] Kurita Y Suzuki A Masuda H Ushiyama T Suzuki K Fujita K. Transition zone volume-adjusted prostate-specific antigen value predicts extracapsular carcinoma of the prostate in patients with intermediate prostate-specific antigen levels. Eur Urol. (1998) 33:32–8. 10.1159/000019532 9471039

[21] Bostwick DG Qian J Bergstralh E Dundore P Dugan J Myers RP. Prediction of capsular perforation and seminal vesicle invasion in prostate cancer. J Urol. (1996) 155:1361–7. 10.1097/00005392-199604000-00064 8632575

[22] Azzouzi AR Vincendeau S Barret E Cicco A Kleinclauss F van der Poel HG. Padeliporfin vascular-targeted photodynamic therapy versus active surveillance in men with low-risk prostate cancer (CLIN1001 PCM301): an open-label, phase 3, randomised controlled trial. Lancet Oncol. (2017) 18:181–91. 10.1016/S1470-2045(16)30661-1 28007457

[23] Wilt TJ Vo TN Langsetmo L Dahm P Wheeler T Aronson WJ. Radical prostatectomy or observation for clinically localized prostate cancer: extended follow-up of the prostate cancer intervention versus observation trial (PIVOT). Eur Urol. (2020) 77:713–24. 10.1016/j.eururo.2020.02.009 32089359

[24] Ward JF Jones JS. Focal cryotherapy for localized prostate cancer: a report from the national Cryo On-Line Database (COLD) registry. BJU Int. (2012) 109:1648–54. 10.1111/j.1464-410X.2011.10578.x 22035200

[25] Mendez MH Passoni NM Pow-Sang J Jones JS Polascik TJ. Comparison of outcomes between preoperatively potent men treated with focal versus whole gland cryotherapy in a matched population. J Endourol. (2015) 29:1193–8. 10.1089/end.2014.0881 26058496

