# Supplementary figures and images for: Comparing the Oncological Outcomes of Cryoablation vs. Radical Prostatectomy in Low-Intermediate Risk Localized Prostate Cancer
Source: Front Oncol. 2020 Aug 26;10:1489. doi: 10.3389/fonc.2020.01489 (PMC7479211; doi:10.3389/fonc.2020.01489)

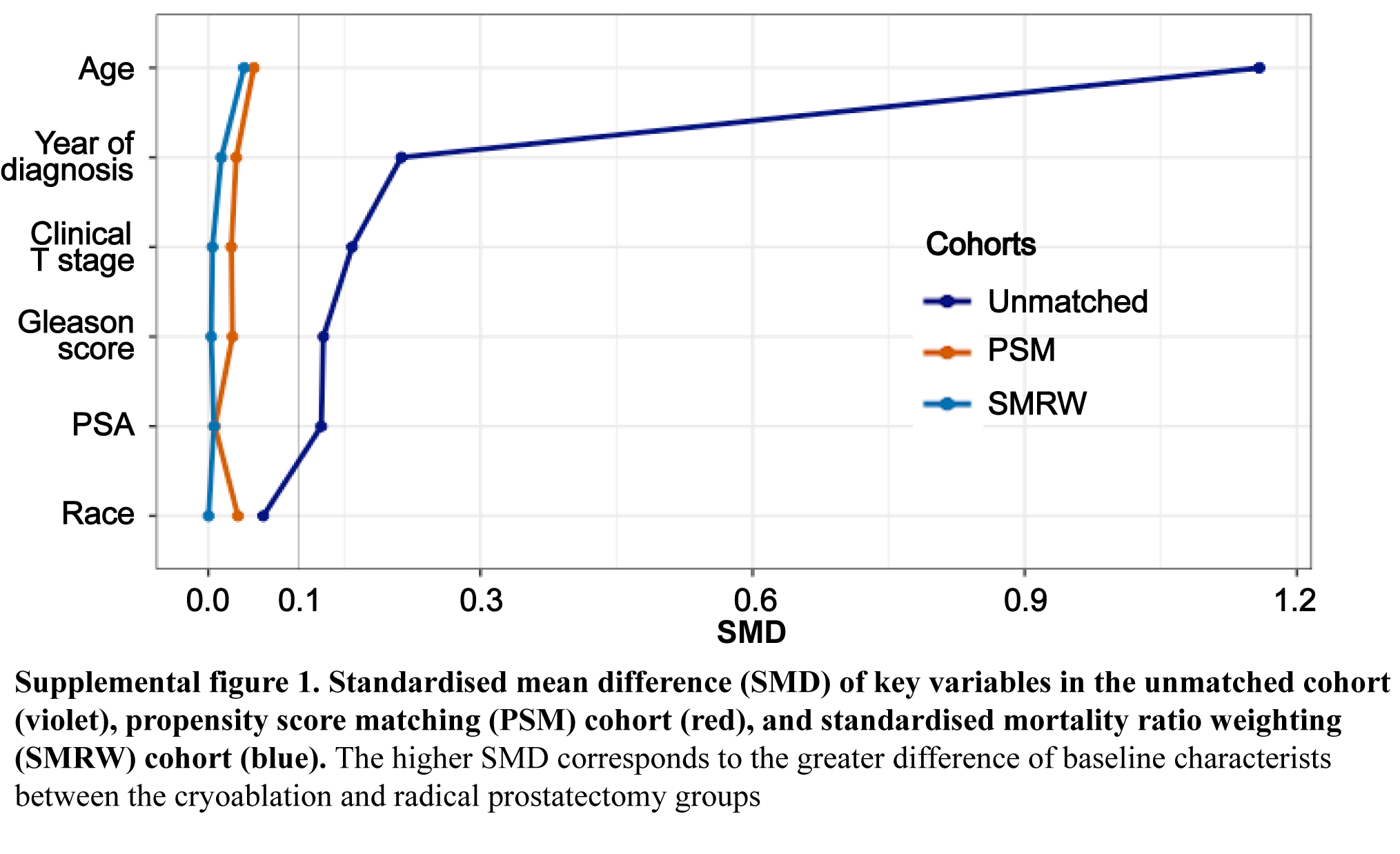

Supplement: Supplementary file 1 [file Image_1.TIF]

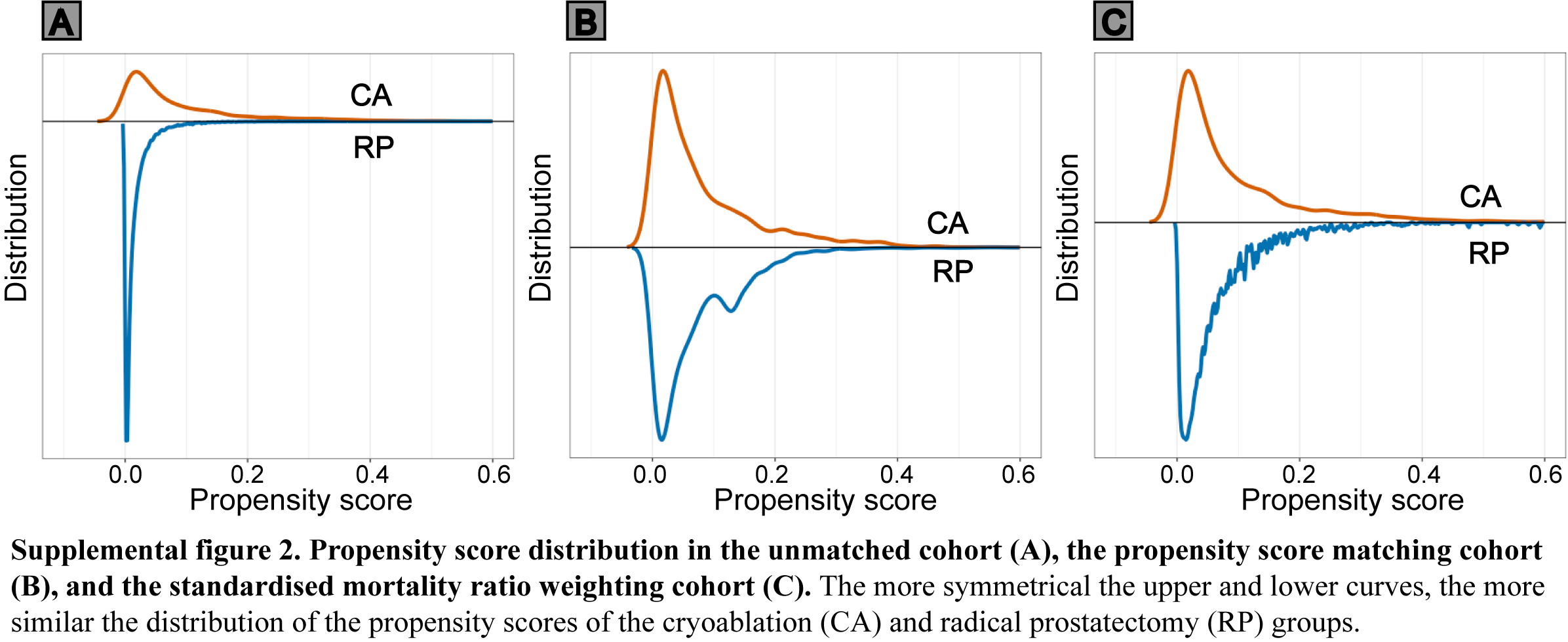

Supplement: Supplementary file 2 [file Image_2.TIF]

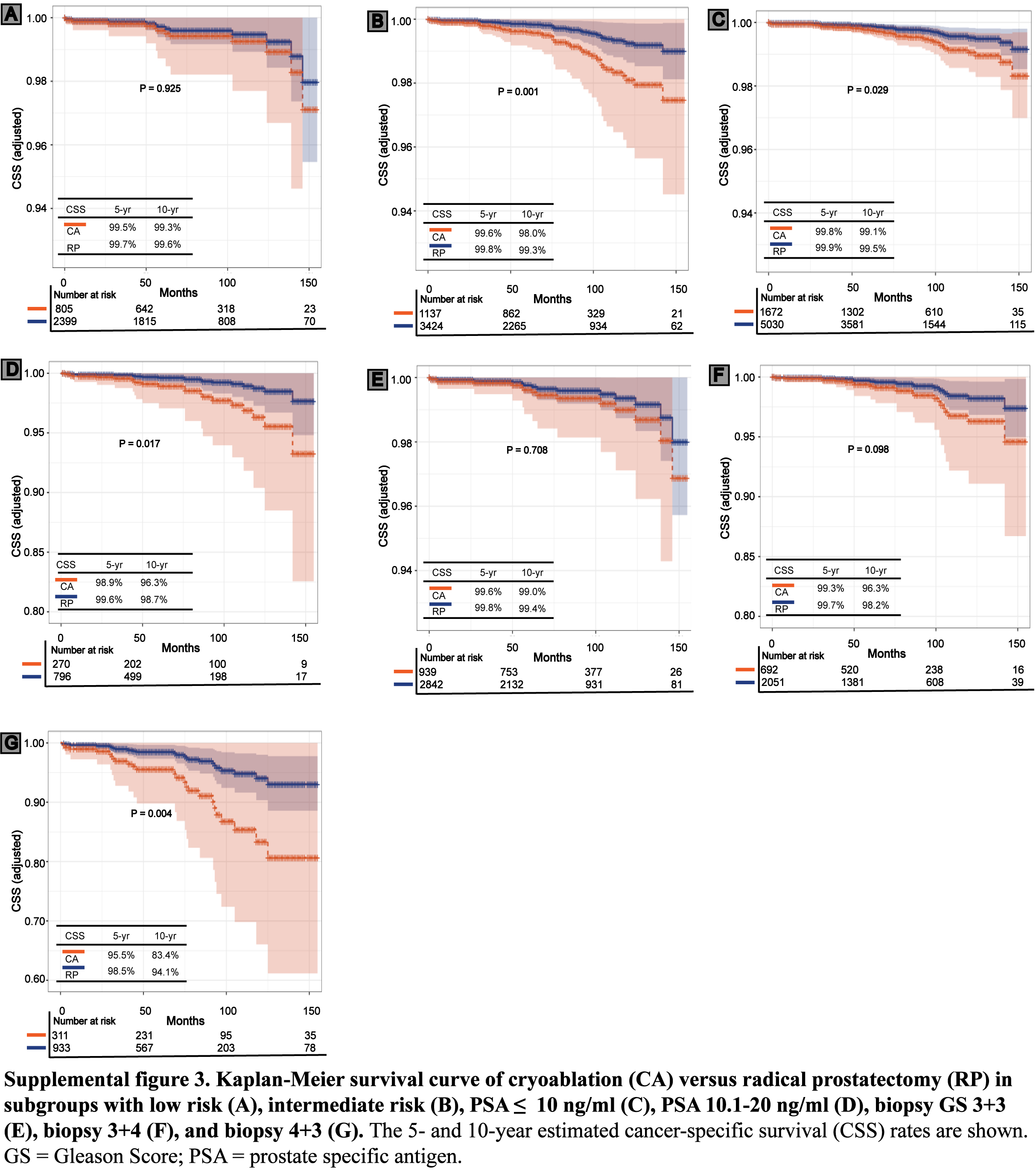

Supplement: Supplementary file 3 [file Image_3.TIF]
